# Structure‐Response Relationships in Rigid *C*_2_‐Symmetric Excitonic Systems: Principles, Modulation, and Functional Design Strategies

**DOI:** 10.1002/cphc.202500712

**Published:** 2026-01-25

**Authors:** Iván Gómez‐Oya, Julia Portela‐Pino, Ani Ozcelik, José Lorenzo Alonso‐Gómez

**Affiliations:** ^1^ Department of Organic Chemistry University of Vigo Vigo Spain; ^2^ Department of Physical Chemistry University of Vigo Vigo Spain

**Keywords:** *C*_2_ symmetry, electron circular dichroism, exciton chirality, *g*‐factor, transition dipole moments

## Abstract

Exciton coupling model provides one of the most intuitive and powerful frameworks to directly connect molecular structure with chiroptical responses. This review focuses on rigid architectures with *C*_2_ symmetry, in which conformational rigidity, symmetry constraints, and independent chromophores allow for direct correlations among molecular geometry, Davydov splitting, and electronic circular dichroism intensity. After introducing the theoretical basis of exciton coupling and its crucial role in absolute configuration assignment, we analyze how molecular design strategies control the conformational space, as well as how the electron transition dipole moments of interacting chromophores enable the modulation of dissymmetry factors (*g*‐factors). Next, we expand these principles from isolated molecules to supramolecular assemblies, thin films, and polymers, where cooperative effects and new structural constraints can come into play to amplify or distort excitonic signatures. Overall, this review compiles transferable design principles to guide the development of next‐generation chiroptical materials with broad relevance for sensing, optoelectronic, and spintronic applications.

## Graphical abstract

Rigid *C*_2_‐symmetric chromophores reveal how transition dipole orientation, magnitude, and conformational locking govern exciton coupling and *g*‐factor amplification, providing a structural blueprint for the rational design of advanced chiroptical molecular and material architectures.

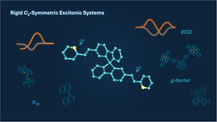

## 1 Introduction

**FIGURE 1:**
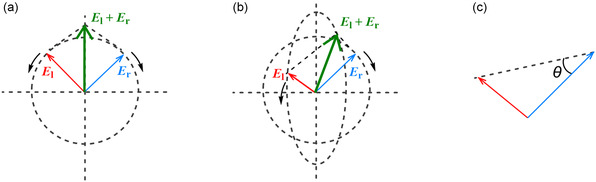
Front view of linearly polarized light (green arrow) represented as the sum of right‐ (blue) and left‐CP components (red), (a) before and (b) after passing through a sample exhibiting CD, and (c) illustration of the resulting ellipticity (*θ*), defined as the deviation of the polarization from linear to elliptical.

Chirality is one of the essential organizing principles of matter. It is defined as the property by which an object or a molecule is not superimposable with its mirror image, giving rise to two forms called enantiomers. This characteristic, originating from the spatial arrangement of atoms, can confer unique optical, electronic, and catalytic properties on molecules. The property of chirality is intrinsically present in biomolecules, such as DNA, proteins, or sugars, and has been deliberately implemented in the design of numerous functional materials with advanced properties [1, 2, 3, 4]. Depending on the symmetry and spatial arrangement of a compound, chirality can manifest itself in different ways. The most common is due to the presence of a stereogenic center, as occurs in the asymmetric carbons of sugars and amino acids. It can also originate from a chiral axis, as encountered in allenes [5] or in binaphthyls [6] or from a plane of chirality, characteristic of certain metal complexes and macrocyclic molecular setups [7]. In all these cases, the assignment of the absolute configuration of different chiral elements follows the Cahn–Ingold–Prelog (CIP) rules, which allow unambiguous assignment of (*R*)/(*S*), (*P*)/(*M*), (*R*a)/(*S*a) or other notations according to a particular chiral element. Chirality, however, is not limited to molecules: light itself can also be chiral. Light is an electromagnetic wave composed of perpendicular oscillating electric and magnetic fields. For chiroptical responses, the electric field component of light is typically considered, which can oscillate linearly or rotate helically. In the latter case, the wave is referred to as circularly polarized (CP) light: if the rotation is clockwise, it is right‐handed CP light, whereas if it is counterclockwise, it is left‐handed CP light. When a chiral sample absorbs differently right‐ and left‐handed CP light, the phenomenon is known as circular dichroism (CD) (Figure 1) [8].

**FIGURE 2:**
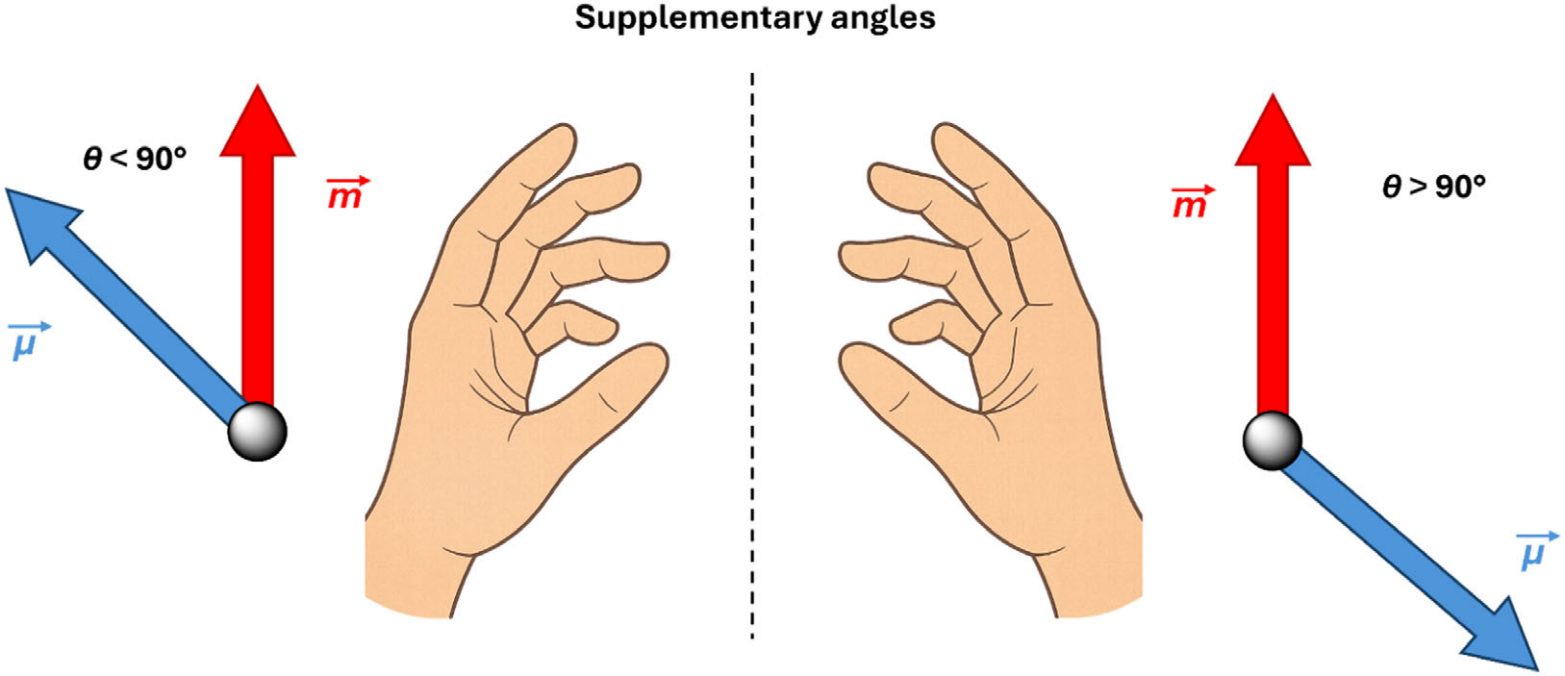
Schematic representation of the relative orientation between the electric (ETDM, *μ*, blue) and magnetic (MTDM, *m*, red) transition dipole moments in a pair of enantiomers. The *θ* supplementary angles lead to rotatory strengths of equal magnitude but opposite sign, resulting in mirror‐image ECD spectra.

For the experimental scrutiny of chiroptical properties, one of the most widely used techniques is electronic circular dichroism (ECD), which specifically probes electronic transitions, as opposed to vibrational CD, VCD addressing vibrational modes of chiral molecules. The physical origin of ECD lies in the so‐called rotatory strength (*RS*):

(1) RS=|μ||m|cosθ

where *μ* is the electric transition dipole moment (ETDM) and *m* is the magnetic transition dipole moment (MTDM) associated with a particular electronic transition. Both the intensity and sign of each band therefore depend on the magnitude of *μ* and *m* and, most importantly, on their mutual orientation. Specifically, under mirror reflection, *μ* reverses its direction (polar vector) while *m* remains unchanged (axial vector). As a result, in a pair of enantiomers, the angles between *μ* and *m* become supplementary. This means that if the angle of an enantiomer is *θ*_R_, then in the mirror‐image enantiomer becomes *θ*_L_ = 180° – *θ*_R_. Such geometrical relationship explains why enantiomers exhibit ECD spectra that are mirror images of each other, showcasing bands of equal magnitude but opposite sign (Figure 2).

Because of the abovementioned vectorial relationship between ETDM and MTDM, ECD is a highly powerful and sensitive toolkit to probe molecular asymmetry. Moreover, it provides a practical quantitative descriptor: the dissymmetry factor, or *g*‐factor, defined as the ratio between the differential absorption (Δ*A*) and the total absorption (*A*) given in:

(2) $$g=\frac{\text{Δ}A}{A}$$

This dimensionless parameter enables a standardized comparison of chiroptical response intensities across different molecules from different hierarchical levels and has become a key metric for evaluating the efficiency of chiroptical responses in the design of advanced chiroptical materials [9, 10].

Beyond its fundamental interests, molecular chirality acts as a strategic and important resource when disclosing new functional material designs. In light–matter interactions, the ability to generate and modulate intense chiroptical responses opens up new and exciting avenues for optoelectronics, enantioselective detection, information processing, and bioimaging applications, where *g*‐factor is used as a comparative reference metric of chiroptical efficiency across systems with different absorption strengths. Similarly, in the field of molecular electronics, chirality plays a decisive role through the effect of chirality‐induced spin selectivity (CISS). Predicted theoretically and experimentally demonstrated in the last decade in organic systems, biomolecules, and self‐assembled films [11], this effect is based on the remarkable ability of chiral molecules to act as spin filters, preferentially transmitting electrons of one spin orientation without the need for external magnetic fields. This property not only challenges conventional paradigms of spin physics but also positions chirality as a functional element capable of bridging optics and electronics. It opens the door to chiral spintronics, molecular computing, and new strategies for energy conversion [12]. Recent studies have correlated the magnitude of CISS with the *g*‐factor, emphasizing that strategies to enhance *g*‐factor values are essential for the rational design of efficient chiroptical systems [13].

In general, the rationalization of ECD responses arises from the prediction of electronic transitions using quantum‐chemical approaches, most commonly time‐dependent density functional theory (TD‐DFT) [1]. Any chiral system can display chiroptical responses, yet a particularly intuitive and widely applied framework is provided by the exciton coupling model, which describes how electronic excitations localized in two or more chromophores interact through space [14, 15]. Exciton coupling gives rise to characteristic bisignate ECD signals, pairs of positive and negative Cotton effects, directly underpinning the relative orientation of the interacting chromophores. Thus, this approach becomes especially powerful for structural elucidation [16]. Because of its intuitive nature, the exciton model has found broad applications in fields, ranging from conformational analysis of biaryl systems to tailored‐made designs of supramolecular architectures. To establish robust structure–response relationships toward high‐performance chiroptical platforms, the combination of conformational rigidity and *C*_2_ symmetry, together with the presence of independent chromophores, provides an ideal framework. Here, rigidity minimizes conformational freedom and thus averaging effects, while *C*_2_ symmetry reduces the description of the system to two well‐defined states. Independent chromophores ensure that the observed ECD couplets arise predominantly from exciton coupling, rather than being obscured by extended delocalization or charge–transfer contributions. Based on the exciton coupling, these structural characteristics enable a direct correlation between molecular geometry and chiroptical intensity, making them particularly powerful tools for the design of advanced chiroptical systems. Notably, in our recent work we have exploited such concept in the development of *C*_2_‐symmetric excitonic systems, using it as a practical and experimentally validated strategy to boost chiroptical responses and enhance *g*‐factors. This demonstrates how rational structural design can be directly translated into amplified chiroptical effects [17].

The aim of this review is to stress the theoretical grounds and structural design principles ruling over exciton coupling in rigid *C*_2_ systems, and to illustrate how these concepts can be harnessed for the development of next‐generation chiral functional materials. To this end, we first outline the theoretical foundations of exciton coupling and its crucial role in rationalizing ECD. Next, we examine rationale behind why specifically *C*_2_ symmetry represents the simplest and most informative scenario of excitonic analysis, showcasing representative examples of rigid *C*_2_‐symmetric systems with different modulation strategies. Finally, we discuss emerging directions and design principles that exploit exciton coupling to boost chiroptical performance in advanced molecular materials.

## 2 Exciton Coupling in ECD: Theoretical Basis

Historically, the concept of coupled electronic oscillators originates from the work of Kuhn in the 1920s and 1930s, who introduced the dipole–dipole interaction model to explain optical rotation dispersion and early CD phenomena [18]. Several decades later, Mason and coworkers provided the first experimental demonstrations of these principles in coordination complexes, establishing exciton coupling model as a microscopic origin of ECD [19]. This framework was subsequently transformed into a versatile stereochemical method through the pioneering studies of Nakanishi and Harada [14], where the authors applied and refined exciton coupling across a broad range of organic and natural products. In the following decades, Berova and collaborators systematized and expanded the approach, integrating it into the chiroptical analysis of complex molecular, supramolecular, and biomolecular architectures [1, 20, 21]. Together, the contributions laid the foundation for the modern Exciton Chirality Method (ECM), which remains a central link between molecular geometry and ECD spectral signatures.

**FIGURE 3:**
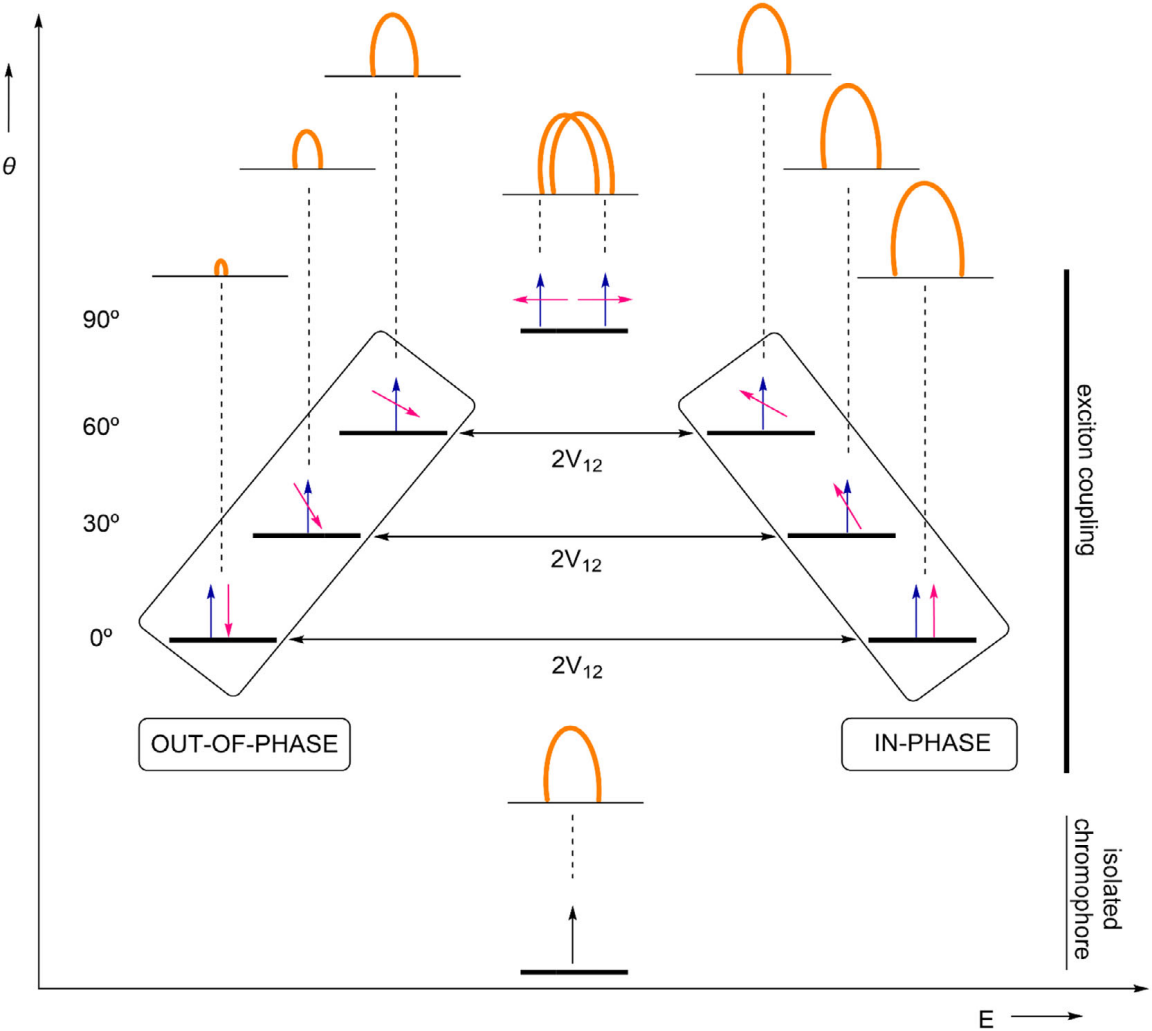
Schematic representation between an isolated (black) and two interacting chromophores (blue and magenta) by exciton coupling. The different torsion angles between the chromophores lead to a splitting of the excitation energy with different relative intensities and Δ*E.* The impact of exciton coupling on the UV/Vis spectra is demonstrated by orange curves.

Building upon these early concepts, a particularly relevant case affecting the intensity of the ECD signal is the phenomenon known as excitonic coupling. When two chromophores with allowed electronic transitions are close enough to interact through space, their ETDMs (*μ*_1_^t^ and *μ*_2_^t^ vectors, respectively) are coupled by a dipole–dipole interaction. This interaction splits the original transition into two excitonic states: an in‐phase state, where the transition dipoles couple in a parallel‐like fashion, and an out‐of‐phase state, where the dipoles couple in an antiparallel‐like fashion. This energetic difference between the two excitonic states is referred as Davydov splitting, and it can be expressed as: Δ*E* = 2*V*_12_ where *V*_12_ is the interaction energy between the two interacting chromophores (Figure 3) [22]. From the spectroscopic point of view, this phenomenon manifests itself in the ECD spectrum as the characteristic exciton couplet, composed of two bands of opposite sign and comparable magnitudes. The shape of these signals reflects the interaction between the transition electric dipoles, providing key structural information about the relative arrangement of the chromophores [9, 10].

Particularly, the appearance and shape of the couplet are directly governed by three fundamental geometric factors: (i) the magnitude of the *μ*_1_ and *μ*_2_, (ii) the distance between the chromophores (*R*_12_), and (iii) the relative orientation of the dipoles, determined by the specific twist between the interacting chromophores. To describe this interaction quantitatively, the Davydov model offers a formulation based on the dipole–dipole tensor and the relative geometry of the chromophores. Therefore, the *V*_12_ can be expressed as:

(3) $$V_{12}=\frac{1}{R_{12}^{3}}\left[\vec{\mu_{1}^{t}}\cdot \vec{\mu_{2}^{t}}-3\frac{\left(\vec{\mu_{1}^{t}}\cdot \vec{R_{12}}\right)\left(\vec{\mu_{2}^{t}}\cdot \vec{R_{12}}\right)}{R_{12}^{2}}\right]$$

According to this model, the coupling energy (*V*_12_) increases with the modulus of the ETDMs (*μ*_1_^t^ and *μ*_2_^t^) and decreases with the interchromophoric distance (*R*_12_) and depends on the angles that define its spatial arrangement. In cases where the ETDM of both chromophores are identical, this expression can be reformulated with the magnitudes as follows, based on the law of proportionality *V*_12_ ∝ *μ* ^2^/*R* ^3^:

(4) $$V_{12}=\frac{\mu^{2}}{R^{3}}\left(\cos\;\theta_{12}-3\cos\;\theta_{1}\cos\;\theta_{2}\right)$$

In rather flexible *C*_2_ frameworks, residual torsional freedom around the symmetry axis renders an exciton couplet strongly dependent on the dihedral angle between chromophores [23]. From a coupled‐dipole perspective, the couplet sign inverts upon reversing the sign of the torsion angle. The observed ECD profiles thus correspond to a Boltzmann‐weighted spectra of lowest energy conformers. This explains why rather flexible *C*_2_ analogs often display less pronounced or even vanishing exciton couplets unless the conformational freedom is locked.

**FIGURE 4:**
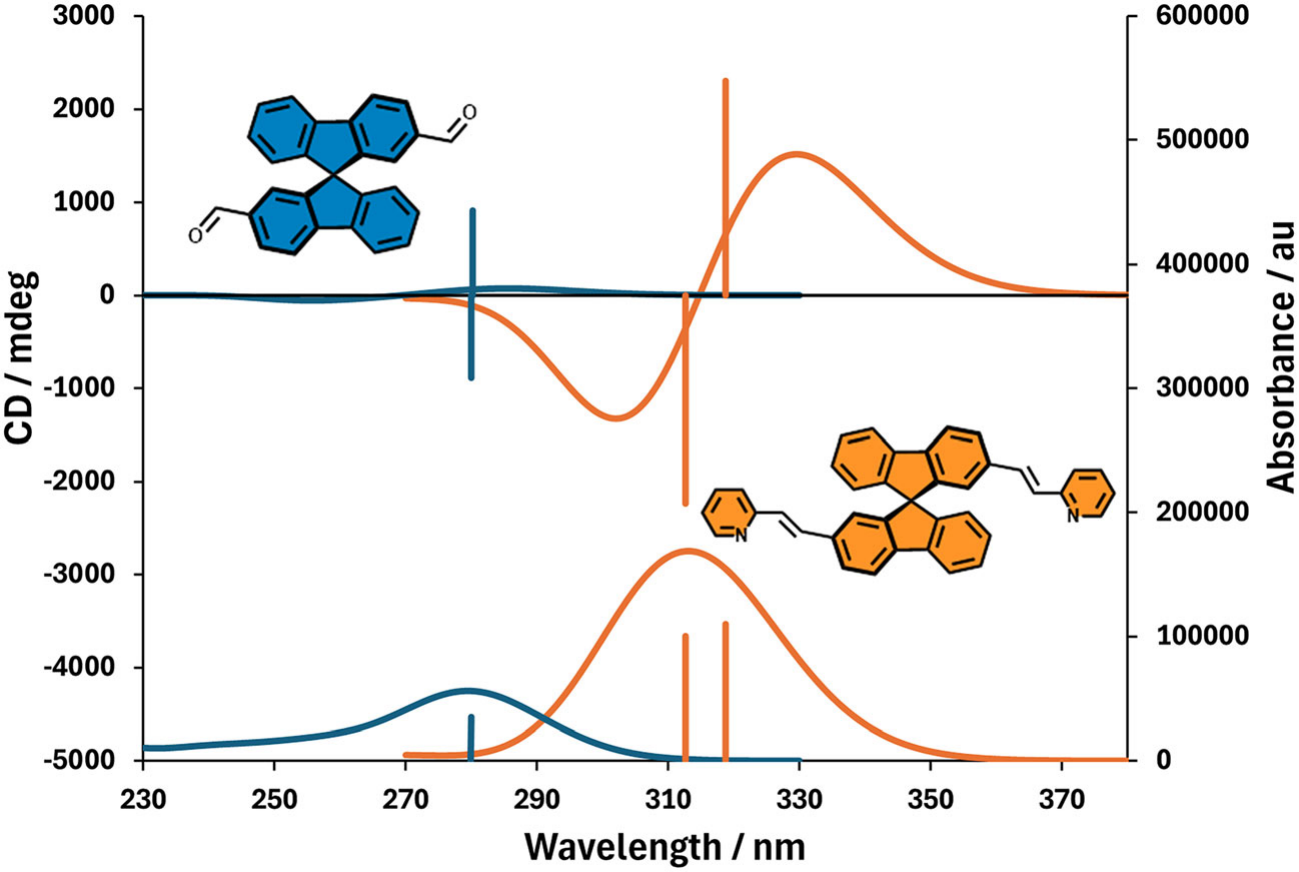
Theoretical ECD (top) and UV–vis (bottom) spectra of spirobifluorene systems exhibiting exciton coupling, depicted by smaller (blue) and larger (orange) *V*_12_ values, computed at the CAM‐B3LYP/6‐31G(d, p) level of theory. For the smaller *V*_12_ (blue), the positive and negative Cotton effects from the in‐phase and out‐of‐phase transitions nearly cancel each other. In contrast, when *V*_12_ is larger (orange), such cancelation effect becomes less pronounced, leading to an enhancement in chiroptical intensity [17]. Theoretical spectra correspond to the trans (*E*) configuration of the 2‐pyridyl‐vinylene substituents. A hypothetical *Z*‐configured (cis) isomer would present reduced conjugation and, consequently, weaker exciton coupling due to a steric‐induced twisting geometry.

The three‐dimensional geometry of the molecule controls the Δ*E* excitonic splitting, as well as the intensity and profile of the ECD signals [16]. These parameters ultimately govern ECD intensity and *g*‐factor value, making the exciton model a key tool for chiroptical analysis of complex molecular systems [16, 22]. However, when dealing with complex systems, in which multiple transitions or more than two chromophores are present, the two‐oscillator model may be insufficient. In such cases, more general approaches are used, such as the DeVoe model, which considers the contribution of multiple oscillators [24], or the Chiroptical Symmetry Analysis (CSA), which connects molecular symmetry with the selection rules to predict ECD spectra [14, 16]. More recently, we have experimentally demonstrated how these principles can be exploited by a tailored molecular design: in rigid *C*_2_ architectures based on spirobifluorene, the introduction of peripheral groups increasing |*μ*| in each subunit reinforces *V*_12_. As a consequence, Δ*E* increases, reducing the cancelation effect between opposite bands in the ECD spectrum and leading to a significant enhancement of the *g*‐factor (Figure 4) [17]. It is also worth noting that in systems bearing 2‐pyridyl‐trans‐vinylene (2PtV) substituents, the extent of conjugation between the pyridyl and fluorene *π*‐systems depends on the C=C double‐bond configuration. The trans (*E*) isomer can adopt a nearly coplanar geometry, allowing efficient *π*‐delocalization and stronger exciton coupling, whereas the cis (*Z*) isomer introduces steric hindrance that twists the pyridyl ring out of plane, weakening conjugation and thus decreasing the ECD couplet intensity. Although the present simulations correspond to the trans configuration, in line with the NMR characterization, this qualitative behavior would account for potential spectral changes upon photoinduced double bond isomerization. A similar geometrical dependence has been extensively characterized for biaryl systems such as 1,1‘‐binaphthyls, where the *syn* and *anti* conformers give rise to couplets of opposite sign, and the excitonic splitting disappears dihedral angles close to 90°, corresponding to orthogonal transition dipoles. This behavior has been rationalized through combined experimental and computational studies (ZINDO/TDDFT) by Di Bari and Pescitelli, confirming that conformational torsion directly governs the magnitude and sign of exciton coupling [6, 25, 26]. This example illustrates how structural design strategies relying on exciton chirality modulates the chiroptical response in a rational manner.

**FIGURE 5:**
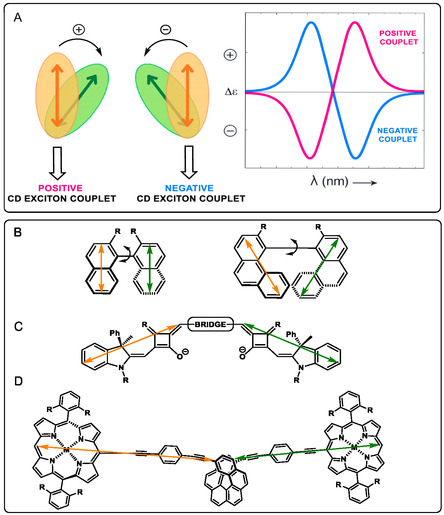
(A) Representation of the ECM (left) and its impact on positive/negative CD exciton couplet in ECD spectra (right). The ETDMs of both chromophores are depicted in orange and green colors. (B) Binaphthyl and biphenanthryl units, (C) squaraine, and (D) helicene‐porphyrins as examples of compounds following the ECM.

Therefore, the theoretical framework of excitonic coupling in ECD directly connects molecular geometry with the spectroscopic response and offers a solid foundation for understanding the variations in intensity and sign observed in spectra, which has led to the development of the ECM. This method follows a nonempirical criterion, serving for the determination of absolute configurations in multichromophoric systems. The method is restricted to chiral systems containing at least two chromophores capable of undergoing electric dipole‐allowed electronic transitions, such as *π*–π* excitations in aromatic, polyenic, or enone‐based units. These chromophores must be spatially close to enable through‐space coupling, while the remaining must be electronically independent, that is, not conjugated with each other nor involved in charge–transfer transitions. ECM builds upon geometry and depends on the helicity (direction of rotation) formed by the ETDMs of the interacting chromophores when they are transferred into space. If the pair of vectors describes a right‐handed helix, the Cotton effect associated with the lowest electronic transition is positive and the subsequent Cotton effect is negative. An opposite negative‐positive Cotton effect trend is true for a left‐handed helicity (Figure 5a) [10]. However, the validity of this analysis requires three basic conditions: (i) the molecular conformation is well defined, (ii) the directions of the ETDMs are known or can be estimated by electronic structure calculations, and (iii) the excitonic coupling is the dominant origin of the observed signal [9, 10]. The validity of ECM has been experimentally confirmed in different systems. Recent examples in squaraine dimers [27] and helicene‐porphyrin conjugates [28] have shown intense couplets, where magnitude and sign strictly follow the helicity and relative arrangement of the chromophores, confirming their applicability in these emerging architectures (Figure 5b–d). Historically, binaphthyls have served as model systems: by varying the torsion angle between the rings, the relative orientation of the ETDMs changes and, in this way, the sign and intensity of the couplet, clearly illustrating the relationship between geometry and chiroptical response [6, 29, 30].

On the other hand, there are nonclassical cases of splitting, in which the sequence of bands does not follow the expected rule due to the mixing with charge transfer states, heavy atom effects, or additional interactions. In this respect, a typical example is 2,2′‐diiodine‐1,1′‐binaphtyl with the UV region of the spectrum, which underlies the crucial need to always verify that excitonic coupling is the dominant mechanism before applying the ECM [29]. It is also worth mentioning that excitonic chirality has its vibrational analog: two IR oscillators (e.g., two C=O) are coupled to generate a couplet in VCD, and its sign follows the same helicity rule, useful when the UV–vis chromophore is missing or not very suitable for ECD [31, 32].

Overall, exciton coupling provides a direct bridge between geometry and spectrum, and ECM constitutes a robust criterion both to assign absolute configurations and rationalize intensity variations. This conceptual framework will be the key to understanding the molecular design strategies discussed in the following sections.

## 3 Chromophore Identity and Molecular Architecture

Within the framework of exciton coupling, a chromophore is defined as a molecular unit bearing a conjugated electronic system capable of absorbing light and undergoing well‐defined electronic transitions. Chromophores are fundamentally responsible for the dipolar interactions that give rise to exciton couplets observed in ECD spectra. Prototypical examples include extended *π*‐systems, such as naphthyl and phenanthryl units, heteroaromatic systems like indolenines, or highly polarizable chromophores, e.g., squaraines and merocyanines, in which intense *π*–π* transitions enable the precise detection of chiroptical responses with high spectral resolution [6, 33, 34]. Crucially, however, aromaticity alone is not sufficient to define an effective chromophore in this context. Structural rigidity is equally important, as only conformationally stable units allow for the reliable prediction of transition dipole orientations. Rigidity preserves the relative geometry of the interacting chromophores, thereby affording stronger and more reproducible couplings with a clear structure‐spectroscopy correlation, while conformational flexibility tends to average out or suppresses the excitonic effect.

Once the chromophore has been established, the way that two such absorbing units are connected within a molecular framework dictates both the magnitude and the sign of the coupling. In acyclic systems, where both chromophores are fused by a C—C bond, such as binaphthyls or biphenanthryls, rotation about the biaryl axis introduces significant conformational freedom, with the dihedral angle *α* between the aromatic planes governing helicity and thus the observed CD signature. Conformations with *α* < 90° typically bear one helicity, whereas those with *α* > 90° produce its mirror‐image helicity, such that the coexistence of multiple conformers in solution accounts for the diversity and, in some cases, attenuation of CD signals (Figure 5B) [6, 30, 33]. In contrast, architectures with rigid bridges restrict their torsional flexibility and impose a fixed spatial relationship between the chromophores, eliminating conformational dispersions and allowing direct correlations between molecular geometry and spectral response (Figure 5C). Helicenes stand as a representative case, where the inherent helical topology of the extended aromatic scaffold enforces axial chirality and produces intense, robust exciton couplets (Figure 5D) [28].

On the other hand, the conservative or nonconservative nature of exciton coupling is likewise governed by above mentioned structural features. The classical exciton model (conservative exciton coupling) predicts a strictly conservative couplet, consisting of two CD bands of equal intensity and opposite sign appearing close to the main electronic transition. Such behavior is strictly observed in rigid and well‐defined architectures, including *C*_2_‐symmetric binaphthyls and helically organized squaraine dimers, where dipole–dipole coupling constitutes the dominant contribution [34, 35]. Yet, many systems do not obey this idealized scheme. Additional electric–magnetic coupling contributions, coplanar geometries that reduce effective chirality, or vibronic coupling effects may distort the intensity balance, and hence produce nonconservative spectra. Recent studies on benzobisthiazole‐bridged dimers have clearly shown that in such cases the excitonic term is ruled out by electric–magnetic interactions, giving rise to asymmetric couplets and spectral signatures that deviate from the classical model [27]. Another notable exception is found in highly organized natural systems. Recent studies on LH1 (bacteriochlorophylls in *Rhodospirillum rubrum*) reveal that, as the number of coupled chromophores increases, both the intensity distribution and the ECD spectral shape stabilize, requiring an extended exciton chirality model to describe the coupling that extends to about 12 units [36]. Consequently, exciton coupling conservativity manifests itself most clearly in rigid, helically arranged systems with well‐isolated chromophores, whereas electronically more complex architectures demand more sophisticated theoretical treatments to rationalize their chiroptical behavior.

## 4 Structure–Property Relationships in Excitonic Chiroptical Systems

The exciton model not only allows for interpreting spectra, but also it offers a direct guidance to amplify chiroptical responses. As mentioned above, the magnitude of excitonic coupling depends on the relative orientation between the electric transition dipole moment (*μ*) of the interacting chromophores and the dimensions of these ETDMs. These parameters govern *V*_12_, Davydov splitting, and *g*‐factor, and any influence on these produces measurable changes in couplet profile and |Δ*ε*|. Therefore, systematic evaluation of these “chiroptical levers” with respect to the *g*‐factor offers powerful means to unravel structure–property relationships in chiroptical systems.

**FIGURE 6:**
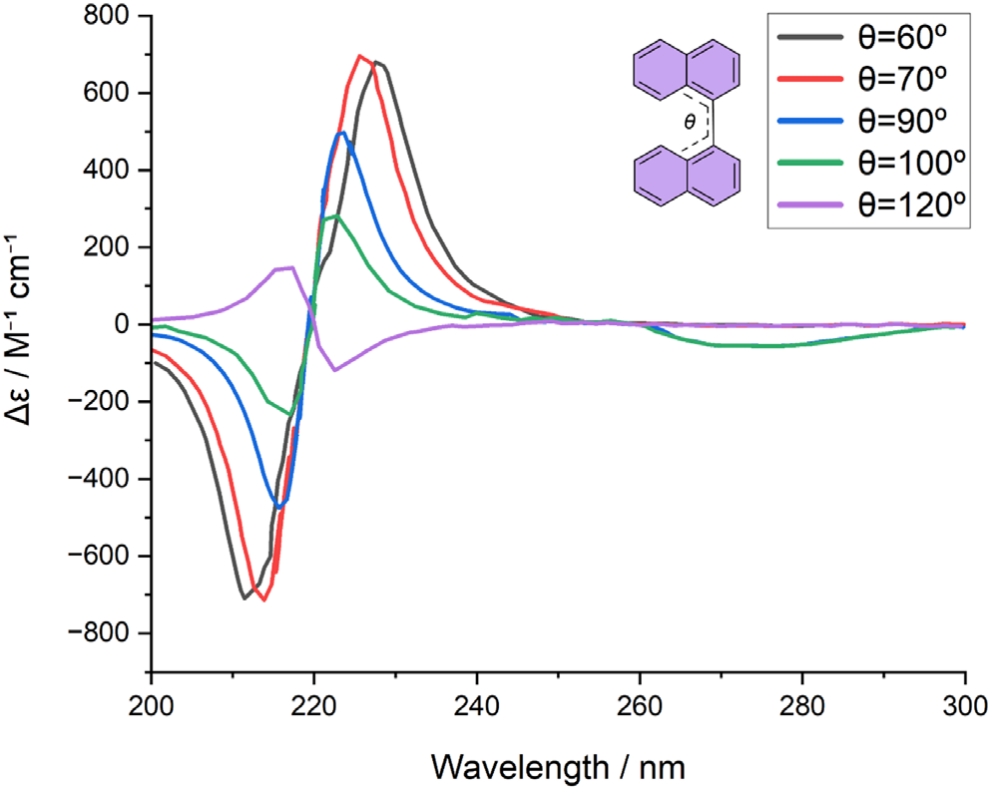
Calculated ECD spectra of (*S*)−1,1′‐binaphthyl as a function of the inter‐ring dihedral angle (*θ*). Progressive torsion modulates both the sign and intensity of the exciton couplet. Cancelation of opposite‐sign contributions occurs at *θ *≈ 90°, in which the transition dipoles become orthogonal.

A first and fundamental lever is structural rigidification. As discussed in the previous section, conformational flexibility averages out distinct orientations of *μ*, which in practice results in an apparent reduction of *V*_12_ and, consequently, in attenuated or even canceled excitonic ECD signals. Locking the relative geometry, by contrast, eliminates such averaging effect and maximizes the efficient coupling. Squaraine dimers bridged by benzobisthiazoles clearly illustrates this principle: the rigid linker fixes the arrangement of the chromophores, optimizes the coupling angle, and yields *g*‐factors in the order of 10^−3^, exceptionally high for organic dimers of this type [27]. In general, rigid frameworks suppress conformational averaging effects and maintain a well‐defined mutual orientation of the interacting transition dipoles. This reinforcement of the coupling term minimizes cancelation between contributions of opposite sign and ultimately produces larger values and stronger chiroptical responses. This behavior is in contrast to flexible biaryl systems, where torsional fluctuations modulate both the sign and amplitude of the exciton couplet and can even eliminate the splitting in conformations almost presenting orthogonal arrangement (Figure 6) [24].

**FIGURE 7:**
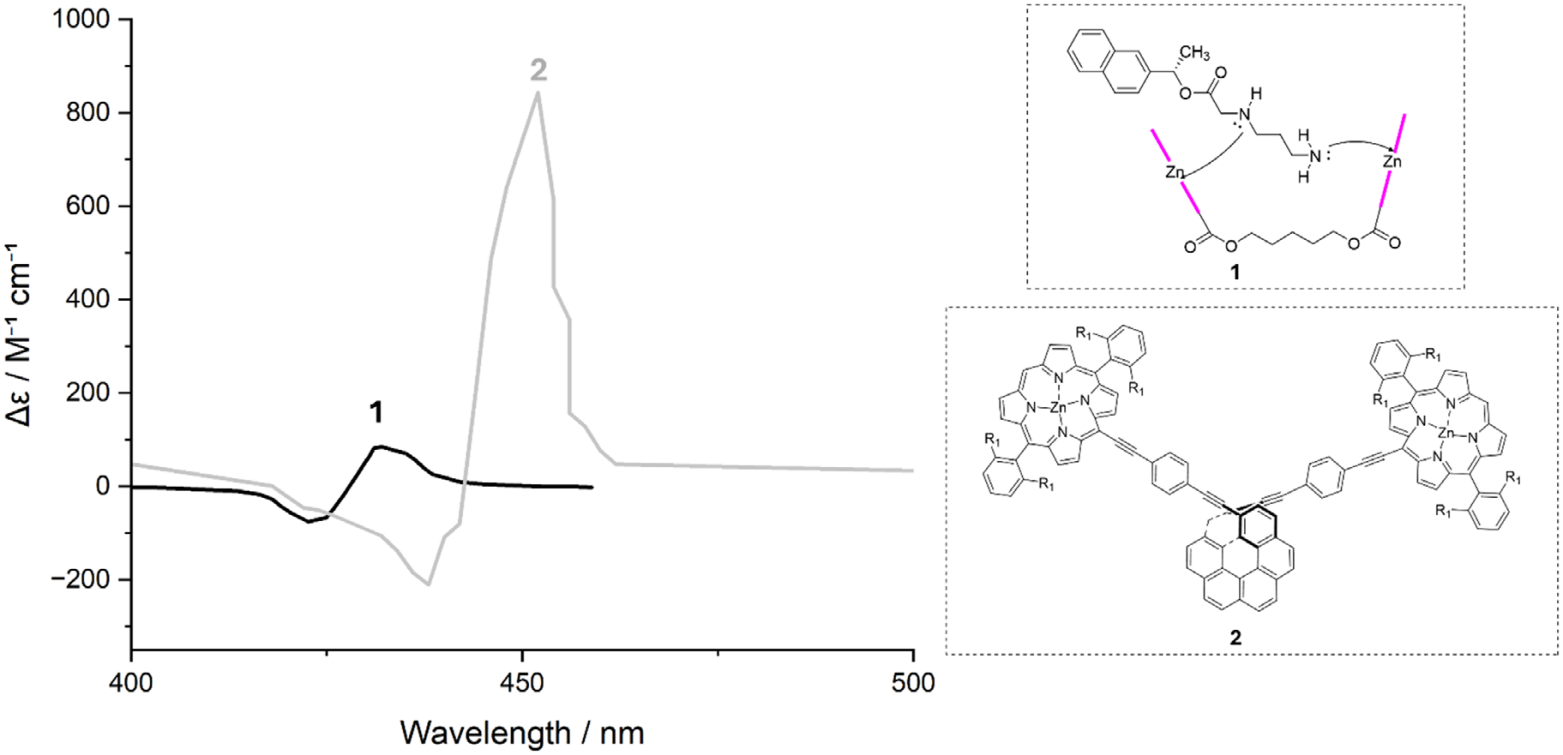
Comparison between helicene‐porphyrin conjugates and porphyrin‐based molecular tweezers. While the flexible tweezers display poorly defined orientations and correspondingly weak ECD responses (black), the rigid helicene backbone enforces a defined orientation of the ETDM vectors and yields intense bisignate couplets in the Soret band (gray). Data and structures adapted from refs. [28, 32].

A second, complementary approach focuses on orienting the ETDM vectors through rational design strategies. While rigidity fixes the relative positions of the chromophores, substituents, *π*‐extension, or the introduction of heteroatoms can reorient *μ*, optimizing excitonic interactions. An illustrative example is the helicene–porphyrin conjugates: the helicene backbone enforces a rigid three‐dimensional framework, while the porphyrins provide intense and highly directional ETDM components. In the region of the Soret band (400–450 nm), these systems display isolated bisignate couplets with very high intensity (Δ*ε* ≈ 6.8 × 10^2 ^M^−1 ^cm^−1^), demonstrating the efficiency of electronic control of *μ* [28]. This performance contrasts sharply with that of porphyrin‐based molecular tweezers, where the relative orientation of the chromophores is poorly defined, and hence the resulting ECD signals are weak [21]. The present comparison highlights that strong chiroptical responses require both rigid scaffolds and precise dipole orientation, and that enhancing |*μ*| alone is insufficient unless combined with structural control (Figure 7) [21, 28].

**FIGURE 8:**
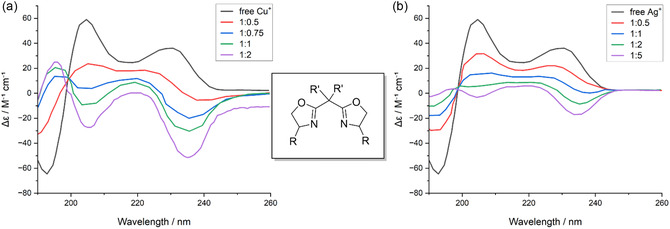
ECD spectra of bis(oxazoline) ligands upon coordination with (a) Cu^+^ and (b) Ag^+^. The free ligands (black) display a weak Cotton effect, while successive addition of metal ions (colored traces) reorganizes the dipole orientation and can invert the sign of the main Cotton effect, highlighting the electronic control imposed by coordination [37].

In a complementary way, metal coordination offers another mean to reorganize the dipoles. In bis(oxazoline) ligands with *C*_2_ symmetry, the coordination to metals such as Cu^+^ or Ag^+^ shows how metal junction not only freezes the conformation, but also it reorganizes the electronic arrangement of the chromophores, even pushing beyond by inverting the sign of the main Cotton effect, the characteristic band of ECD associated with a given electron transition [37]. In all these cases, the key point is that fixing the geometry alone is not sufficient to boost the ECD signal and transition vectors must be electronically directed toward more favorable configurations (Figure 8) [37].

**FIGURE 9:**
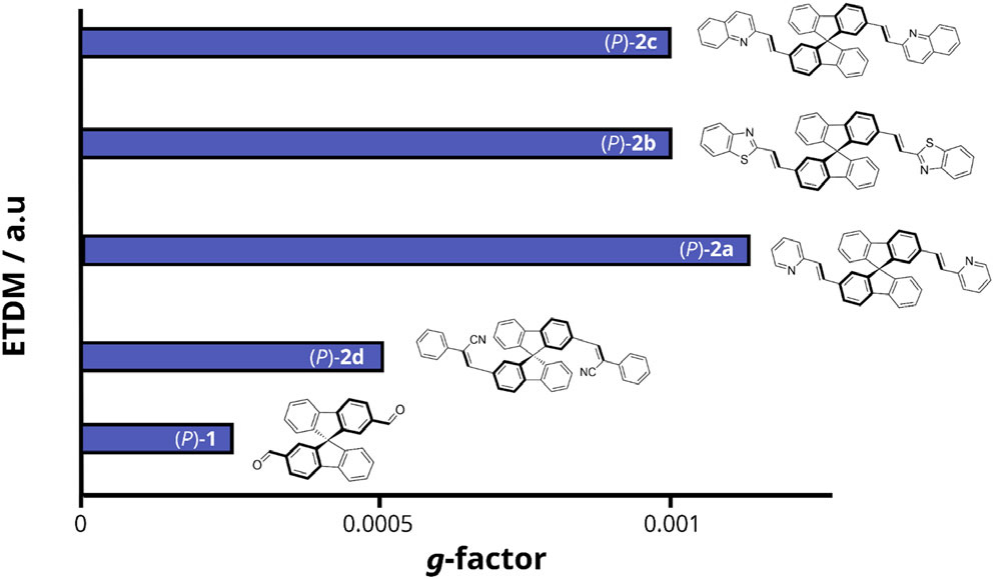
Correlation between the magnitude of the electric transition dipole moment (|*μ*|) of interacting chromophores and the *g*‐factor in *C*_2_‐symmetric SBF derivatives, where π‐extension enhances |*μ*| and leads to a systematic amplification of chiroptical responses [17].

A third and particularly illustrative strategy is to amplify the magnitude of the transition dipoles once their relative orientation is defined. Because excitonic interaction is proportional to |*μ*|^2^ (*V*_12_ ∝ |*μ*|^2^), enhancing local oscillator strength increases *V*_12_, the Davydov splitting, and reduces cancelation effects between transitions, as illustrated in Figure 4. We have recently demonstrated that this principle in *C*_2_‐symmetric SBF derivatives substituted at the 2,2′‐positions: *π*‐extended fragments introduced by Knoevenagel condensations systematically increased the modulus of *μ* in each subunit, while the relative orientation remained almost fixed. Our study highlights a direct correlation between |*μ*| and the *g*‐factor, with experimental evidence of fivefold chiroptical amplification (Figure 9) [17].

Taken together, these examples showcase that CD intensity can be rationally modulated through three complementary mechanisms: structural rigidification to suppress the conformational average, control on the orientation of the ETDM if interacting chromophores are present, and amplification of *μ* to directly strengthen exciton coupling. While *C*_2_ symmetry provides a particularly clear and easily tailorable framework to analyze these effects, the underlying design principles are general and can be extended to other molecular architectures with more than two interacting chromophores [22]. In this sense, the combination of conformational control, optimal orientation of ETDM vectors, and tunable ETDM magnitude constitutes a robust design platform for next‐generation chiroptical materials. An important question arises at this point: to what extent do these principles are valid when molecules organize at higher length scales, in aggregates, films, or polymers, where new structural constraints and collective phenomena can either amplify or distort exciton coupling?

## 5 From Molecular Design to Chiroptical Materials

The rational design of chiroptical materials requires a clear translation from molecular‐level parameters to macroscopic responses. The exciton model, originally formulated to describe the coupling of electronic transitions in molecular aggregates, has emerged as a common ground for this purpose. By connecting molecular descriptors, such as electric and magnetic transition dipole moments, their relative orientation, and conformational rigidity for collective optical properties, the exciton model provides a powerful bridge between chemical structure and material function. Recent studies have demonstrated that even slight variations in transition dipole strength or chromophore arrangement can dramatically alter the *g*‐factor, highlighting how molecular “chiroptical levers” can be systematically tuned to maximize performance [17]. Extending this logic from discrete molecules to supramolecular assemblies and solid‐state materials opens up new avenues toward the predictive design of next‐generation chiroptical platforms [16].

From discrete *C*_2_‐symmetric molecules, the next step involves *π*‐conjugated oligomers where exciton coupling becomes cooperative along extended helicoidal backbones. Recently, Uceda et al. reported fully *π*‐conjugated helical architectures combining rigid [6]helicenes and flexible o‐OPE linkers, achieving *g*_abs_ ≈ 2 x 10^−2^. Since circularly polarized luminescence (CPL) is the differential emission of left‐ and right‐handed CP light and it can be quantified by the luminescence dissymmetry factor (*g*_lum_ = 2(*I*_*L*_ – *I*_*R*_)/(*I*_*L*_ + *I*_*R*_)) analogous to *g*_abs_ in ECD, the resulting chiroptical luminophore afforded a remarkable CPL response with *g*_lum_ ≈ 1 x 10^−2^. DFT/MD simulations confirmed that conformational locking and *μ*/*m* alignment govern their enhanced chiroptical performance [38]. In parallel, Bræstrup et al. synthesized helical ladder oligomers based on annulated [6]helicene‐PDI units and observed super‐linear growth of CPL brightness with chain length due to progressive dipole alignment and exciton delocalization [39]. At a similar level, recent work on stereochemically controlled *π*‐conjugated polymers has demonstrated that a rigid backbone and a defined helical bias can dictate both the magnitude and the sign of exciton coupling, with reported |*g*| values approaching the 10^−1^ regime [40]. These examples illustrate the transition from isolated chromophores to cooperative *π*‐extended frameworks, where exciton coupling becomes an invaluable design variable for both absorption and emission responses.

At the polymer level, the transition from molecular to material scale can be achieved by programing the helicity of the main chain. A representative case involves ladder‐type polymers based on SBF, featuring rigid polymeric skeletons built through multiple linkages that restrict torsional motion. In these materials, the programable helicity enforces cooperative excitonic coupling along the entire chain, leading to intense and stable ECD spectra and CPL emissions with significantly higher *g*_lum_ values than those attainable for isolated molecules [41]. These results demonstrate how the principles discussed above—molecular stiffness, *μ* orientation, and the magnitude of |*μ*|—can be transferred to the design of polymeric materials with robust chiroptical responses.

At the supramolecular scale, self‐assembly into excitonic aggregates provides another strategy for next‐generation chiroptical materials. In these systems, the Frenkel‐type electron excitation, normally confined to one chromophore, becomes delocalized across multiple units owing to intermolecular proximity. When molecules are stacked in a head‐tail fashion, forming so‐called J‐aggregates with well‐defined morphology, the spectral bands become narrower and redshifted. If the aggregate also adopts a helical twist, supramolecular chirality arises and generates amplified ECD signals. When fluorophores are incorporated, this collective order can also enhance CPL. While in isolated molecules *g*_lum_ rarely exceeds 10^−3^, values approaching 0.08 have been reported for helical aggregates, demonstrating how supramolecular organization can amplify the chiroptical response [42].

In condensed phases, molecular proximity can be further exploited in thin films or surface‐adsorbed layers, where chromophores interact more strongly than their solution‐based congeners. This often results in increased absorption anisotropy (*g*_abs_), sometimes fivefold higher than in dilute conditions [43]. However, such films pose experimental challenges: measured ECD spectra can be distorted by linear dichroism (LD) or by linear birefringence (LB) when the sample is not perfectly isotropic. To correct these artifacts, techniques such as Mueller‐matrix polarimetry are employed to isolate true chiral contributions from anisotropic effects, ensuring that the excitonic coupling signal is genuine [43]. Similar issues have been identified in nematic solvents, where partial chromophore orientation introduces misleading LD/LB signals. Quantifying an order parameter (S) or applying alignment controls allows distinction between real chiroptical activity and false anisotropy [44]. These precedents show that accurate interpretation of ECD in organized materials requires as much experimental care as molecular design.

Beyond molecular films, early demonstrations of chirality transfer to macroscopic optical responses include noncentrosymmetric thin films of helicene–bisquinone derivatives exhibiting strong second‐harmonic generation (*χ* ^2 ^≈2–9 pm V^−1^) without external poling, i.e., without applying an electric field to orient dipoles at the macroscopic scale [45]. In this case, the intrinsic molecular chirality itself provides the required noncentrosymmetry for a measurable *χ* ^2^ response, analogous to how *g*_lum_ quantifies chiral selectivity in CPL. This classical example illustrates how molecular handedness can induce measurable nonlinear optical activity solely through self‐assembly, consolidating the concept of exciton‐mediated amplification in solid‐state architectures.

A third strategy of chirality transfer at the material scale is found in cholesteric liquid crystals, where a small amount of chiral dopant induces a helical twist throughout the matrix. The pitch of this helical structure determines both optical selectivity and chiral signal intensity. In such systems, the ECD response reflects not only the excitonic interactions between individual molecules but also the helical organization of the material as a whole. Thus, rational molecular design yields strongly amplified chiroptical signatures when chirality is transferred and hierarchically organized within the cholesteric phase [46].

Beyond molecular and supramolecular emission, exciton‐coupling concepts have been successfully extended to device‐scale photonic architectures. In hybrid and supramolecular CPL detectors, cooperative exciton coupling and anisotropic charge transport have yielded remarkably high photocurrent dissymmetry factors (*g*_ph_) approaching 0.6, demonstrating that chiroptical selectivity can be transduced into an electronic signal [47]. Moreover, embedding chiral emitters into cholesteric liquid‐crystal polymer networks or perovskite nanostructures produces photonic Bragg reflection that selectively amplifies one circular polarization, leading to *g*_lum_ values exceeding 10^−1^ [48]. These examples confirm that macroscopic optical activity can be rationally engineered by transferring molecular control of *μ*/*m* orientation to ordered material phases, bridging excitonic and photonic scales.

Beyond static structural effects, exciton coupling also governs the photophysical regime of emission. According to Kasha's rule, fluorescence typically originates from the lowest excited singlet state (S_1_); however, in strongly coupled or delocalized systems, radiative decay may occur from higher excitonic levels (S_2_ → S_0_), resulting in anti‐Kasha behavior [49, 50]. Such cases often display reversed or unexpectedly enhanced CPL fingerprints relative to ECD, since the transition‐dipole orientation and delocalization differ between the two excitonic states [51]. Recognizing this interplay between exciton coupling and Kasha/anti‐Kasha regimes is essential for interpreting chiroptical emission in aggregates and conjugated polymers.

Overall, the progression from molecular to macroscopic scales reveals how the same exciton‐coupling principles underpin the entire hierarchy of chiroptical materials. From the precise control of *μ* and *m* orientation in discrete chromophores to their cooperative alignment in oligomers, polymers, and ordered assemblies, chirality can be rationally translated into measurable optical anisotropy and circular polarization. Excitonic amplification thus emerges as the unifying mechanism that bridges chemical design and device function, linking the microscopic determinants of geometry and transition dipoles with the macroscopic manifestation of chiral light–matter interactions.

## 6 Summary and Outlook

In this review, we have showcased the path that goes from the theoretical foundations of exciton coupling to its application in rigid molecular architectures with *C*_2_ symmetry and, ultimately, in supramolecular and polymeric materials. This review reveals a common message: the intensity of the excitonic ECD can be understood and enhanced if key structural parameters are identified and controlled, as well as they are coherently transferred from the molecular to the material scale.

Among the structural determinants that govern a strong excitonic coupling are the magnitude of the electric transition dipole moments (|*μ*|) of interacting chromophores, their relative orientation (*θ*), the torsion and slipping between chromophores, and the presence of stiffness elements that prevent the conformational averaging effects. When combined with simple symmetries like *C*_2_, these factors reduce the complexity of the spectrum and enable direct correlations between geometry and chiroptic response [10, 16]. It is also essential to maintain the independence of chromophores, avoiding excessive delocalization or mixing with charge transfer states, which can weaken or distort the excitonic torque [29].

From these principles, concrete molecular design guidelines can be extracted: structural stiffening using covalent bridges or bicyclic architectures [27], modulation of *μ* orientation by substituents, functionalization, or metal coordination [28, 37], and the reinforcement of the magnitude |*μ*| by conjugate fragments or Knoevenagel condensation as useful synthetic toolkit [17]. These strategies are not independent, but act synergistically: a successful design usually involves synergies of rigidity, favorable orientation, and intense electric transition dipole moments.

As shown in Sections 4 and 5, these principles operate at different scales: at the molecular level (control of *μ*, *θ* and rigidity), at the supramolecular level (programing helical or cooperative packing), and at the macroscopic level (e.g., orientation in films and measurement protocols). This multiscale progress illustrates how translating concepts from isolated molecules to chiral materials paves the way for optoelectronic devices, sensors, and spintronics platforms, where chirality is no longer a mere structural feature but an active and tunable functional design element [2]. Thus, the study of rigid excitonic systems not only provides a solid theoretical framework to interpret ECD, but also it lays the foundational ground for a new generation of chiroptical materials with impacts across science and technology.

## Funding

This study was supported by MCIU/AEI/FEDER/10.13039/501100011033 (TED2021‐131760B‐100), MCIU/AEI/FEDER/10.13039/501100011033(PID2023‐146155OB‐I00), Consellería de Economía, Emprego e Industria, Xunta de Galicia (ED431C 2017/51) and Consellería de Cultura, Educación e Ordenación Universitaria, Xunta de Galicia (ED431F 2023/090 : Funding for open accesscharge : Universidade de Vigo/CRUE‐CISUG).
